# Supplementary material for: Multichannel speech enhancement for automatic speech recognition: a literature review
Source: PeerJ Comput Sci. 2025 Mar 27;11:e2772. doi: 10.7717/peerj-cs.2772 (PMC12190416; doi:10.7717/peerj-cs.2772)
Supplement: Supplemental Information 1 [file peerj-cs-11-2772-s001.docx]

**Quality Assessment (QA) Questions**

Quality assessment was carried out using the following QA questions:

**List of Questions:**

| Q1 | Are the aims of the research clearly stated? |
| --- | --- |
| Q2 | Does the article report recent methods and techniques in multi-channel speech enhancement for ASR? |
| Q3 | Any solutions provided towards the formulated RQs? |
| Q4 | Does the article provide results for assessing the performance of multi-channel speech enhancement for ASR? |
| Q5 | Does the article mention challenges and future directions related to the multi-channel speech enhancement for ASR? |

**Assessment scores:**

Each article was subjected to assessment according to the above questions and the articles with scores of 6 and above were selected:

| **No** | **Title** | **Q1** | **Q2** | **Q3** | **Q4** | **Q5** |  |
| --- | --- | --- | --- | --- | --- | --- | --- |
|  |  | **0/1/2** | **0/1/2** | **0/1/2** | **0/1/2** | **0/1/2** |  |
|  |  | ***Aims clearly stated*** | ***ASR Model & Techniques*** | ***Solution provided*** | ***performance*** | ***Challanges and future directions*** | ***Total*** |
| 1 | LEVERAGING REDUNDANCY IN MULTIPLE AUDIO SIGNALS FOR FAR-FIELD SPEECH RECOGNITION | 2 | 1 | 2 | 1 | 1 | 7 |
| 2 | LEARNING MASK SCALARS FOR IMPROVED ROBUST AUTOMATIC SPEECH RECOGNITION | 2 | 2 | 2 | 1 | 2 | 9 |
| 3 | VARARRAY MEETS T-SOT: ADVANCING THE STATE OF THE ART OF STREAMING DISTANT CONVERSATIONAL SPEECH RECOGNITION | 2 | 2 | 1 | 1 | 2 | 8 |
| 4 | REAL-TIME MULTICHANNEL SPEECH SEPARATION AND ENHANCEMENT USING A BEAMSPACE-DOMAIN-BASED LIGHTWEIGHT CNN | 2 | 1 | 2 | 1 | 1 | 7 |
| 5 | Unsupervised Speech Enhancement Based on Multichannel NMF-Informed Beamforming for Noise-Robust Automatic Speech Recognition | 2 | 1 | 2 | 2 | 1 | 8 |
| 6 | Integration of Optimized Modulation Filter Sets Into Deep Neural Networks for Automatic Speech Recognition | 2 | 1 | 1 | 1 | 1 | 6 |
| 7 | DOES SPEECH ENHANCEMENT WORK WITH END-TO-END ASR OBJECTIVES?: EXPERIMENTAL ANALYSIS OF MULTICHANNEL END-TO-END ASR | 2 | 2 | 2 | 1 | 0 | 7 |
| 8 | FLEXIBLE MULTICHANNEL SPEECH ENHANCEMENT FOR NOISE-ROBUST FRONTEND | 2 | 1 | 1 | 1 | 2 | 7 |
| 9 | A Robust Conformer-Based Speech Recognition Model for Mandarin Air Traffic Control | 2 | 1 | 1 | 1 | 1 | 6 |
| 10 | The CHiME-7 Challenge: System Description and Performance of NeMo Team’s DASR System | 2 | 1 | 1 | 2 | 2 | 8 |
| 11 | Non-negative Matrix Factorization Based Noise   Reduction for Noise Robust Automatic Speech   Recognition | 2 | 1 | 2 | 2 | 0 | 7 |
| 12 | Speech enhancement by combining spectral subtraction and minimum mean square error‑spectrum power estimator based on zero crossing | 2 | 1 | 1 | 1 | 2 | 7 |
| 13 | Modeling Speech Structure to Improve T-F Masks for Speech Enhancement and Recognition | 2 | 1 | 2 | 2 | 1 | 8 |
| 14 | PARTIALLY ADAPTIVE MULTICHANNEL JOINT REDUCTION OF EGO-NOISE AND ENVIRONMENTAL NOISE | 2 | 1 | 2 | 2 | 1 | 8 |
| 15 | Automatic Speech Recognition for Indoor HRI Scenarios | 2 | 2 | 2 | 1 | 1 | 8 |
| 16 | Deep Learning Assisted Time-Frequency Processing for Speech Enhancement on Drones | 2 | 1 | 2 | 1 | 1 | 7 |
| 17 | TE-KWS: Text-Informed Speech Enhancement for Noise-Robust Keyword Spotting | 2 | 1 | 2 | 1 | 1 | 7 |
| 18 | An Auditory Inspired Amplitude Modulation Filter Bank for Robust Feature Extraction in Automatic Speech Recognition | 2 | 1 | 0 | 1 | 0 | 4 |
| 19 | PERFORMANCE MONITORING FOR AUTOMATIC SPEECH RECOGNITION IN NOISY MULTI-CHANNEL ENVIRONMENTS | 2 | 2 | 2 | 1 | 1 | 8 |
| 20 | MULTICHANNEL AUDIO FRONT-END FOR FAR-FIELD AUTOMATIC SPEECH RECOGNITION | 2 | 1 | 1 | 1 | 2 | 7 |
| 21 | Multichannel Signal Processing With Deep Neural Networks for Automatic Speech Recognition | 2 | 1 | 2 | 1 | 1 | 7 |
| 22 | Spatial HuBERT: Self-supervised Spatial Speech Representation Learning for a Single Talker from Multi-channel Audio | 2 | 1 | 1 | 1 | 2 | 7 |
| 23 | 3-D ACOUSTIC MODELING FOR FAR-FIELD MULTI-CHANNEL SPEECH RECOGNITION | 2 | 1 | 1 | 1 | 1 | 6 |
| 24 | EFFECT OF NOISE SUPPRESSION LOSSES ON SPEECH DISTORTION AND ASR PERFORMANCE | 2 | 2 | 1 | 2 | 1 | 8 |
| 25 | Should deep neural nets have ears? The role of auditory features in deep learning approaches | 2 | 2 | 2 | 1 | 1 | 8 |
| 26 | Optimizing Integrated Features for Hindi Automatic Speech Recognition System | 2 | 2 | 1 | 1 | 1 | 7 |
| 27 | GFCC based discriminatively trained noise robust continuous ASR system for Hindi language | 2 | 1 | 1 | 1 | 1 | 6 |
| 28 | DUAL APPLICATION OF SPEECH ENHANCEMENT FOR AUTOMATIC SPEECH RECOGNITION | 2 | 2 | 1 | 1 | 0 | 6 |
| 29 | A proposed method to improve the WER of an ASR system in the noisy reverberant room | 2 | 1 | 2 | 2 | 1 | 8 |
| 30 | Space-and-speaker-aware acoustic modeling with effective data augmentation for recognition of multi-array conversational speech | 2 | 1 | 1 | 1 | 1 | 6 |
| 31 | Deep neural network-based generalized sidelobe canceller for dual-channel far-field speech recognition | 2 | 1 | 1 | 1 | 2 | 7 |
| 32 | Deep learning based multi-source localization with source splitting and its effectiveness in multi-talker speech recognition | 2 | 1 | 1 | 1 | 2 | 7 |
| 33 | An artificially intelligent approach for automatic speech processing based on triune ontology and adaptive tribonacci deep neural networks | 2 | 2 | 1 | 1 | 1 | 7 |
| 34 | Train from scratch: Single-stage joint training of speech separation and recognition | 2 | 1 | 1 | 2 | 2 | 8 |
| 35 | Speech enhancement system using deep neural network optimized with Battle Royale Optimization | 2 | 1 | 2 | 1 | 1 | 7 |
| 36 | CNN-based noise reduction for multichannel speech enhancement system with DWT preprocessing | 2 | 1 | 1 | 1 | 2 | 7 |
| 37 | Combining Deep Learning with Domain Adaptation and Filtering Techniques for Speech Recognition in Noisy Environments | 2 | 1 | 2 | 2 | 1 | 8 |
| 38 | Optical Microphone-Based Speech Reconstruction System with Deep Learning for Individuals with Hearing Loss | 2 | 1 | 1 | 1 | 1 | 6 |
| 39 | AUDIO AND VIDEO PROCESSING OF UAV-BASED SIGNALS IN THE HARMONIC PROJECT | 2 | 1 | 2 | 1 | 1 | 7 |
| 40 | Psycho-acoustics inspired automatic speech recognition | 2 | 1 | 0 | 1 | 1 | 5 |
| 41 | Application of dynamic time warping optimization algorithm in speech recognition of machine translation | 2 | 1 | 0 | 1 | 0 | 4 |
| 42 | Emphasizing unseen words: New vocabulary acquisition for end-to-end speech recognition | 2 | 1 | 1 | 1 | 2 | 7 |
| 43 | Joint speaker diarization and speech recognition based on region proposal networks | 2 | 1 | 1 | 2 | 1 | 7 |
