# Supplementary material for: Multichannel speech enhancement for automatic speech recognition: a literature review
Source: PeerJ Comput Sci. 2025 Mar 27;11:e2772. doi: 10.7717/peerj-cs.2772 (PMC12190416; doi:10.7717/peerj-cs.2772)
Supplement: Supplemental Information 2 [file peerj-cs-11-2772-s002.docx]

**Average Word Error Rate (WER) for MCSE and ASR Studies**

The following table shows the average WER scores at different Signal-to-noise ratios (SNR) in this SLR article. The unit of measure for the given values is Percentage (%).

| **Article \|SNR (dB)** | **-12** | **-10** | **-6** | **-5** | **-3** | **0** | **3** | **5** | **6** | **9** | **10** | **12** | **15** | **19** | **20** | **25** | **30** |
| --- | --- | --- | --- | --- | --- | --- | --- | --- | --- | --- | --- | --- | --- | --- | --- | --- | --- |
| (Chang et al., 2023) |  |  |  |  |  | 4.13 |  | 4.875 |  |  |  |  | 9.375 |  |  |  |  |
| (Narayanan et al., 2023) |  |  |  | 17.745 |  | 15.855 |  | 10.616 |  |  |  |  |  |  |  |  |  |
| (Chhetri et al. 2018) | 45.5 |  |  |  |  |  |  |  | 27.5 |  |  |  |  | 17.5 |  |  |  |
| (Sainath et al. 2017) |  |  |  |  |  |  |  |  |  |  |  | 25.4 |  |  |  |  |  |
| (Dimitriadis et al. 2023) |  |  |  |  |  | 9.15 |  |  |  |  |  |  |  |  |  |  |  |
| (Purushothaman, Sreeram, and Ganapathy 2020) |  |  |  |  |  |  |  |  |  |  |  |  |  |  | 13.96 |  |  |
| (Braun and Gamper 2022) |  |  |  |  |  |  |  | 18.0 |  |  |  |  |  |  |  |  |  |
| (Moritz, Kollmeier, and Anemuller 2016) |  |  | 42.05 |  | 34.4 | 28.95 | 23.95 |  | 19.3 | 17.35 |  |  |  |  |  |  |  |
| (Dua, Aggarwal, and Biswas 2019b) |  |  |  |  |  | 52.5 |  | 42.15 |  |  | 29.48 |  | 27.12 |  | 22.87 |  |  |
| (Dua, Aggarwal, and Biswas 2019a) |  |  |  |  |  | 37.0 |  | 30.1 |  |  | 23.1 |  | 19.7 |  | 14.70 |  |  |
| (Jukić, Balam, and Ginsburg 2024) |  |  | 14.36 |  |  | 13.88 |  |  |  |  |  |  |  |  |  |  |  |
| (Pandey et al. 2021) |  |  |  |  |  | 4.52 |  |  |  |  |  |  |  |  |  |  |  |
| (Jiang et al. 2023) |  |  |  |  |  |  | 5.27 |  |  |  |  |  |  |  |  |  |  |
| (Sadeghi, Sheikhzadeh, and Emadi 2024) |  |  |  |  |  |  |  | 32.5 |  |  | 28.9 |  |  |  | 21.7 |  |  |
| (Li et al. 2021) |  |  |  |  |  | 51.7 |  | 36.27 |  |  |  |  |  |  |  |  |  |
| (Subramanian et al. 2022) |  |  |  |  |  | 16.73 |  |  |  |  |  |  |  |  |  |  | 24.63 |
| (Qu, Weber, and Wermter 2023) |  |  |  |  |  |  |  |  |  |  | 8.44 |  |  |  |  |  |  |
| (Shi et al. 2022) |  |  |  |  |  |  |  | 9.50 |  |  |  |  |  |  |  |  |  |
| (Cherukuru and Mustafa 2024) |  | 30.16 |  | 28.18 |  | 21.92 |  | 24.64 |  |  | 24.96 |  | 27.26 |  |  |  |  |
| (Velásquez-Martínez et al. 2023) |  |  |  |  |  | 18.69 |  |  |  |  |  |  |  |  |  |  |  |
| (Kim et al. 2012) |  |  |  |  |  | 70.7 |  | 36.85 |  |  | 22.0 |  | 14.47 |  |  |  |  |
| (Bu et al. 2022) |  |  |  |  |  | 8.46 |  |  |  |  |  |  |  |  |  |  |  |
| (Fang et al. 2023) |  |  |  |  | 59.50 |  |  |  |  |  |  |  |  |  |  |  |  |
| (Novoa et al. 2021) |  |  |  |  |  | 13.76 |  |  |  |  |  |  |  |  |  |  |  |
| (Jokisch et al. 2021) |  |  |  |  |  |  | 4.8 | 6.7 |  |  |  |  |  |  | 0 | 30.4 |  |
| AVE WER | 45.5 | 30.16 | 28.21 | 22.96 | 46.95 | 24.53 | 11.34 | 22.93 | 23.4 | 17.35 | 22.81 | 25.4 | 19.59 | 17.5 | 14.64 | 30.4 | 24.63 |

**Average Perceptual Evaluation of Speech Quality (PESQ) for MCSE and ASR Studies**

The following table shows the average PESQ scores at different Signal-to-noise ratios (SNR) used in this SLR article.

| **Article \|SNR (dB)** | **-7** | **-4** | **-3** | **-1** | **0** | **5** | **10** | **15** | **20** | **25** | **50** |
| --- | --- | --- | --- | --- | --- | --- | --- | --- | --- | --- | --- |
| (Olivieri et al. 2023) |  |  |  |  |  |  |  |  |  |  | 1.72 |
| (Sadeghi, Sheikhzadeh, and Emadi 2024) |  |  |  |  |  | 1.1 | 1.09 |  | 0.84 |  |  |
| (Shukla, Shajin, and Rajendran 2024) |  |  |  |  |  | 1.8 | 1.87 | 1.96 | 2.08 | 2.16 |  |
| (Wang and Cavallaro 2021) | 1.46 |  | 1.77 | 1.59 |  | 1.87 |  |  |  |  |  |
| (Liu et al. 2023) |  | 3.6 |  |  |  |  |  |  |  |  |  |
| AVE PESQ | 1.46 | 3.6 | 1.77 | 1.59 |  | 1.59 | 1.48 | 1.96 | 1.46 | 2.16 | 1.72 |

**Short-Time Objective Intelligibility (STOI) for MCSE-ASR Studies**

The following table shows the average STOI scores at different Signal-to-noise ratios (SNR) used in this SLR article.

| **Article \|SNR (dB)** | **-5** | **0** | **5** | **10** | **15** | **20** | **25** |  |
| --- | --- | --- | --- | --- | --- | --- | --- | --- |
| (Pandey et al. 2021) | 0.698 | 0.785 | 0.698 |  |  |  |  |  |
| (Shukla, Shajin, and Rajendran 2024) |  |  | 0.98 | 0.96 | 0.94 | 0.95 | 0.98 |  |
| AVE STOI | 0.698 | 0.785 | 0.839 | 0.96 | 0.94 | 0.95 | 0.98 |  |

**Performance Scores According to Datasets**

The following table shows the average PESQ, STOI, and WER score comparison for the articles using the same databases for evaluation. These results are depicted in Figures 7, 8, and 9 in this SLR article.

| **Speech dataset** | **Article** | **PESQ** | | | **STOI** | | | **WER** | | | **Method/techniques** |
| --- | --- | --- | --- | --- | --- | --- | --- | --- | --- | --- | --- |
|  |  | **Min** | **Max** | **AVE** | **Min** | **Max** | **AVE** | **Min** | **Max** | **AVE** |  |
| Aurora | (Martinez, Moritz, and Meyer 2014) |  |  |  |  |  |  | 3.9 | 27.9 | 13.6 | AMFB, Observation Adding |
|  | (Cherukuru and Mustafa 2024) |  |  |  |  |  |  | 21.92 | 30.16 | 26.18 | CNN, DWT, Beamforming |
| CHiME | (Purushothaman, Sreeram, and Ganapathy 2020) |  |  |  |  |  |  | 12.9 | 14.4 | 13.7 | S3-D Convolutional Neural Network (CNN) |
|  | (Bu et al. 2022) |  |  |  |  |  |  | 5 | 18.8 | 8.45 | CNN, MVDR Beamformer |
|  | (Shimada et al. 2019) | 2.57 | 2.84 | 2.64 | 0.92 | 0.97 | 0.94 | 6.24 | 15.52 | 11.93 | MWF, MVDR Beamformer, Multichannel NMF |
|  | (Ochiai, Watanabe, and Katagiri 2017) | 1.66 | 1.86 | 1.73 |  |  |  | 12.9 | 14.4 | 12.12 | Mel scale filters, Beamforming |
|  | (Park et al. 2023) |  |  |  |  |  |  | 18 | 54.1 | 38.81 | MVDR Beamformer, Guided Source Separation |
|  | (Chai et al. 2022) |  |  |  |  |  |  | 28.48 | 46.87 | 34.75 | WPE, Mask Based Beamforming |
| Librispeech | (Jiang et al. 2023) |  |  |  |  |  |  | 5.27 | 18.4 | 12.95 | U-net, T5 error correction |
|  | (Narayanan et al. 2023) |  |  |  |  |  |  | 9.5 | 29.3 | 14.73 | Multi-channel Cleanformer, Mask Scalars |
|  | (Cherukuru and Mustafa 2024) |  |  |  |  |  |  | 26.28 | 37.42 | 31.66 | CNN, DWT, Beamforming |
|  | (Qu, Weber, and Wermter 2023) |  |  |  |  |  |  | 5.29 | 14.65 | 8.44 | Multichannel Attention |
|  | (Velásquez-Martínez et al. 2023) | 1.5 | 2.06 | 1.82 | 0.7 | 0.91 | 0.84 | 21.92 | 30.16 | 18.69 | DNN, Wavelet Transform |
|  | (Dimitriadis et al. 2023) |  |  |  |  |  |  | 5.7 | 10.46 | 9.15 | Masked Prediction and denoising |
| TIMIT | (Wang and Cavallaro 2021) | 1.42 | 1.87 | 1.67 |  |  |  |  |  |  | DNN-TF, DNN- BF |
|  | (Dua, Aggarwal, and Biswas 2019a) |  |  |  |  |  |  | 22.87 | 52.5 | 34.83 | HMM-GMM |
|  | (Shukla, Shajin, and Rajendran 2024) | 1.8 | 2.16 | 1.97 | 0.94 | 0.98 | 0.96 |  |  |  | DNN with BRO, Kalman Filter |
|  | (Yadava and Jayanna 2019) | 4.29 | 4.51 | 4.39 |  |  |  |  |  |  | Spectral subtraction with voice activity detection |
|  | (Deepak et al. 2022) |  |  |  |  |  |  | 1.85 | 4.83 | 3.34 | DNN |
|  | (Sadeghi, Sheikhzadeh, and Emadi 2024) | 0.84 | 1.1 | 1.01 |  |  |  | 21.7 | 32.5 | 28.9 | WPE, Super Directive Beamformer |
|  | (Fang et al. 2023) |  |  |  |  |  |  | 45 | 80 | 59.54 | Variational Autoencoder VAE, Multichannel-NMF |
| WSJ | (Shi et al. 2022) |  |  |  |  |  |  | 7 | 15.6 | 9.5 | RNN (LSTM), Multi-source Neural Beamformer |
|  | (Jukić, Balam, and Ginsburg 2024) |  |  |  | 0.62 | 0.74 | 0.7 | 13.7 | 15.19 | 14.12 | Neural mask filter, T-F masking, Mask based Neural Beamformer |
|  | (Subramanian et al. 2022) |  |  |  |  |  |  | 10.6 | 63.1 | 20.68 | MVDR Beamformer |
| Others | (Chang et al. 2023) |  |  |  |  |  |  | 2.0 | 11 | 6.12 | multi-channel acoustic echo cancellation (MCAEC), adaptive beamforming |
|  | (Chhetri et al. 2018) |  |  |  |  |  |  | 17.5 | 45.5 | 30.16 | MVDR, DNN based Beamforming |
|  | (Sainath et al. 2017) |  |  |  |  |  |  | 16.8 | 27.8 | 25.4 | DNN Based Adaptive Beamforming |
|  | (Pandey et al. 2021) |  |  |  | 0.698 | 0.785 | 0.727 |  |  | 4.52 | DCRN |
|  | (Olivieri et al. 2023) | 1.66 | 1.86 | 1.73 |  |  |  |  |  |  | CNN, Ideal ratio Mask, DOA, Super-directive Beamforming |
|  | (Braun and Gamper 2022) |  |  |  |  |  |  | 5.8 | 30.1 | 18 | CLSTM, DNN |
|  | (Moritz, Kollmeier, and Anemuller 2016) |  |  |  |  |  |  | 14.3 | 52.1 | 27.66 | Amplitude Modulation Filter Banks (AMFBs) |
|  | (Dua, Aggarwal, and Biswas 2019b) |  |  |  |  |  |  | 14.7 | 37 | 24.9 | Noise Robust GFCC |
|  | (Li et al. 2021) |  |  |  |  |  |  | 27.34 | 51.76 | 38.4 | MCLP, (DNN)-based Generalized Sidelobe Canceller (GSC) structure, called nnGSC, for beamforming |
|  | (Kim et al. 2012) |  |  |  |  |  |  | 10.2 | 75.8 | 31.25 | NMF, Wiener Filter |
|  | (Novoa et al. 2021) |  |  |  |  |  |  | 4.1 | 39.1 | 13.76 | MVDR Beamformer |
|  | (Jokisch et al. 2021) |  |  |  |  |  |  | 4.8 | 30.4 | 10.47 | Low pass filtering notch filtering, Beamforming |
|  | (Liu et al. 2023) |  |  | 3.6 |  |  | 0.98 |  |  |  | TE-KWS, CADA, Beamforming |

**Performance Vs Noise Type for** MCSE-ASR Studies

The following table shows the overall average WER scores of each article for each type of noise mentioned in Figure 10 of this SLR article. The unit of measure for the given values is Percentage (%).

| Article | Average WER (10%) | Noise type |
| --- | --- | --- |
| (Jokisch et al. 2021) | 10.475 | Ego Noise |
| (Novoa et al. 2021) | 13.76 | Reverberation+ Ego Noise |
| (Moritz, Kollmeier, and Anemuller 2016) | 27.66667 | Reverberation |
| (Jukić, Balam, and Ginsburg 2024) | 14.12 | Reverberation |
| (Sadeghi, Sheikhzadeh, and Emadi 2024) | 27.7 | Reverberation |
| (Li et al. 2021) | 43.985 | Reverberation |
| (Shi et al. 2022) | 9.5 | Reverberation |
| (Narayanan et al. 2023) | 14.73333 | Environmental Noise+ Reverberation |
| (Chhetri et al. 2018) | 30.16667 | Environmental Noise+ Reverberation |
| (Sainath et al. 2017) | 25.4 | Environmental Noise+ Reverberation |
| (Dimitriadis et al. 2023) | 9.15 | Environmental Noise+ Reverberation |
| (Fang et al. 2023) | 59.5 | Environmental Noise+ Ego Noise |
| (Chang et al. 2023) | 6.123333 | Environmental Noise |
| (Shimada et al. 2019) | 11.933 | Environmental Noise |
| (Ochiai, Watanabe, and Katagiri 2017) | 12.12 | Environmental Noise |
| (Deepak et al. 2022) | 3.34 | Environmental Noise |
| (Park et al. 2023) | 38.81 | Environmental Noise + Reverberation |
| (Chai et al. 2022) | 34.76 | Environmental Noise+ Reverberation |
| (Purushothaman, Sreeram, and Ganapathy 2020) | 13.96 | Environmental Noise |
| (Braun and Gamper 2022) | 18 | Environmental Noise |
| (Dua, Aggarwal, and Biswas 2019b) | 34.83 | Environmental Noise |
| (Dua, Aggarwal, and Biswas 2019a) | 24.92 | Environmental Noise |
| (Pandey et al. 2021) | 4.52 | Environmental Noise |
| (Jiang et al. 2023) | 5.27 | Environmental Noise |
| (Subramanian et al. 2022) | 20.68 | Environmental Noise |
| (Qu, Weber, and Wermter 2023) | 8.44 | Environmental Noise |
| (Cherukuru and Mustafa 2024) | 26.18667 | Environmental Noise |
| (Velásquez-Martínez et al. 2023) | 18.69 | Environmental Noise |
| (Kim et al. 2012) | 36.005 | Environmental Noise |
| (Bu et al. 2022) | 8.46 | Environmental Noise |
| (Kanda et al. 2023) | 19.11 | Environmental Noise |

The following table uses the values from the above table to present the overall average WER (%) for each type of noise mentioned in Figure 6 of this SLR article.

|  | EGO Noise | Reverberation | Environmental Noise | Environmental Noise+ Reverberation | Environmental Noise+ Ego Noise | Reverberation+ Ego Noise |
| --- | --- | --- | --- | --- | --- | --- |
| AVE WER | 10.475 | 24.59433 | 16.03459 | 25.50333 | 59.5 | 13.76 |
